# Genetic Markers for Metabarcoding of Freshwater Microalgae: Review

**DOI:** 10.3390/biology12071038

**Published:** 2023-07-22

**Authors:** Elena Kezlya, Natalia Tseplik, Maxim Kulikovskiy

**Affiliations:** Laboratory of Molecular Systematics of Aquatic Plants, K.A. Timiryazev Institute of Plant Physiology RAS, IPP RAS, 127276 Moscow, Russia; ntseplik@gmail.com (N.T.); max-kulikovsky@yandex.ru (M.K.)

**Keywords:** barcode, metabarcoding, ecological assessment, microalgae, genetic markers

## Abstract

**Simple Summary:**

The metabarcoding approach is widely used for studying the diversity and distribution of freshwater microalgae and for routine biomonitoring. Due to microalgae being a phylogenetically diverse group, the choice of a genetic marker directly affects the metabarcoding results. Specific markers are good for identifying only concrete groups, while universal markers may miss classes or lack the variability necessary for differentiating taxa at the species and sometimes genus levels. An analysis of publications on the subject showed that metabarcoding studies of eukaryotic freshwater microalgae used 12 markers (different nuclear regions 18S and ITS and plastid regions *rbc*L, 23S and 16S). Studies that compared outcomes from different markers show that the resulting lists of taxa do not match. The plastid marker *rbc*L is widely used for diatom metabarcoding, as it differentiates taxa at the species and intraspecies levels, and there is a specific set of primers designed for identifying Eustigmatophyceae. The V9 18S region is more variable than V4 18S and provides more diversity at higher taxonomic levels (supergroup and phylum). The ITS1 and ITS2 regions are used rarely and may be underestimated. These barcodes amplify well with the standard primers and are variable enough to identify sequences at the species level. Plastid markers (23S and 16S rDNA) focused on the plastid-containing eukaryotic algae and Cyanobacteria, conserved regions, identify taxa to the genus level and higher. Using specialized curated databases for data interpretation significantly improves the quality of the results.

**Abstract:**

The metabarcoding methods for studying the diversity of freshwater microalgae and routine biomonitoring are actively used in modern research. A lot of experience has been accumulated already, and many methodological questions have been solved (such as the influence of the methods and time of sample conservation, DNA extraction and bioinformatical processing). The reproducibility of the method has been tested and confirmed. However, one of the main problems—choosing a genetic marker for the study—still lacks a clear answer. We analyzed 70 publications and found out that studies on eukaryotic freshwater microalgae use 12 markers (different nuclear regions 18S and ITS and plastids *rbc*L, 23S and 16S). Each marker has its peculiarities; they amplify differently and have various levels of efficiency (variability) in different groups of algae. The V4 and V9 18S and *rbc*L regions are used most often. We concentrated especially on the studies that compare the results of using different markers and microscopy. We summarize the data on the primers for each region and on how the choice of a marker affects the taxonomic composition of a community.

## 1. Introduction

Currently eDNA metabarcoding is a popular method for studying the diversity and functioning of various communities, from microbes to mammals. Interest in this method grows every year, and the number of studies increases. For example, a query in the SCOPUS database with the keyword “metabarcoding” returns 2215 results; a query with the keyword “eDNA” returns 26,034 results (date of search 12 February 2023).

Algae are a phylogenetically heterogeneous group of organisms that is very diverse in morphology and ecological preferences. In the eukaryotic tree of life, photosynthetic eukaryotes are spread across 12 separate phylogenetic lines at the level of phylum [1,2,3]. On a macrosystematic level, they belong to four to seven (according to different estimates) supergroups that also contain non-photosynthetic organisms in each clade [1,2,3,4,5,6]. This phylogenetical heterogeneity is connected with a gene locus “…which is variable enough to provide robust identification at the species level…” [7] (and references in it) “…and different markers are applied for species delimitation in different algal groups.” [8] (and references in it). For example, phylogenetic studies and species descriptions of diatoms and red algae do not use the rDNA ITS marker, whereas it is the main marker currently employed for DNA-based species of green microalgae, Dinoflagellates, Chrysophytes and Synurophytes [7,8] (and references in it). Well-documented nucleotide sequences are accumulated in databases, which are the basis of interpreting the metabarcoding data.

Thus, the choice of the barcode region and primer pairs, which can limit or bias the diversity of organisms observed, is a challenge with environmental metabarcoding studies [9,10]. The proportion of biodiversity covered by metabarcoding studies directly depends on the markers and primers used, so organisms that are not amplified by standard methods go undetected, even if they are common and play an important role in the ecosystem [11]. It is important for a “good barcode” to be taxonomically informative; it needs to be able to distinguish between species (i.e., the DNA region should mutate at the right rate), because most modern biomonitoring and biotic index programs require identification at the species level. At the same time, a barcode needs conserved primer binding areas, or degenerate primers, in order to be able to attach to the DNA of all the organisms in the sample [12]. The choice of primers also impacts the results of a biodiversity assessment of an ecosystem. Complete universality causes a loss of resolution and limits the depth of the biodiversity assessments of groups. Limiting the universality of the primers might, on the other hand, exclude important groups in the analysis and introduce biases, favoring some organisms or groups. Furthermore, the use of different universal primers makes direct comparisons between studies more challenging [13] (and references in it). All these conditions show that metabarcoding is not a simple and universal method of monitoring and biodiversity studies of algae, as it has its limitations, and further development and tuning are needed. The choice of marker also plays an important role in the interpretation of results.

There is a lot of experience already gained in using next-generation sequencing (NGS) approaches for studying algae. One of the high-priority research areas is the integration of metabarcoding into routine biomonitoring. Many methodological questions have been answered; bioinformatics pipelines have been assessed [14,15], sampling, DNA extraction methods and applications of global eDNA have been discussed [16,17,18,19,20,21], and recently, it has been shown that the preservation time and sample preservation methods have little effect on DNA metabarcoding results [22], the experience of integrating eDNA metabarcoding into routine freshwater biomonitoring has been summarized [23,24,25] and the terminology “eDNA” has been clarified [26]). In a recent study, Salmaso et al. [27] looked into the problem of a taxonomic gap in reference databases for aquatic cyanobacteria and eukaryotic microalgae and the effect it has on the interpretation of metabarcoding data. Extensive reviews of the methodology of DNA metabarcoding in marine bulk samples have been published, including benthic communities of sediment and hard substrate, plankton samples and dietary samples [12] and freshwater harmful algae *Microcystis aeruginosa* and *Prymnesium parvum* [28]. The state of DNA barcoding of macroalgae in the Mediterranean Sea has also been reviewed [29].

In metabarcoding studies dedicated to freshwater eukaryotic algae, various genetic markers (nuclear regions 18S V3, V4, V4–V5, V7, V7–V9, V9, V9-ITS1 and ITS2 and plastid regions *rbc*L, 16S and 23S) and various primer sets have been used (Table 1). The aim of this review is to summarize the available information and to critically assess which markers and primers are the most effective for metabarcoding freshwater algae, how they should be chosen, what the level of taxonomic coverage and resolution is and which databases are used for the taxonomic attribution of sequences.

## 2. Materials and Methods

The search for literary sources was carried out in the SCOPUS database in February 2023 using the keywords “metabarcoding”, “algae”, “markers”, “barcode”, “freshwater”, “eDNA”, “diatom”, “protist”, “NGS”, “18S”, “ITS”, “23S”, “*rbc*L” and “16S” in combinations of two or three keywords. The results of each search were reviewed. The selection of appropriate publications was conducted according to the following criteria: (1) the research article or review was published in a peer-reviewed journal, (2) the research concerned freshwater algae or eukaryotic organisms in general, (3) the study examined the results of metabarcoding and (4) genetic markers were discussed. In total 70 studies published in the period from 2013 to 2023 were analyzed. Also, 5 studies of marine microalgal communities were included in the review in the section that discussed the comparison of metabarcoding results based on the V4 and V9 18S regions.

Simple histograms for a graphic representation of the obtained results were constructed using MS Excel. The list of publications used in analysis is provided in Table S1 (Supplementary Materials). The list of genetic markers and primer sets is provided in Table 1. The sets of primers were assigned conditional numbers for convenience (numbered in order for each region).

## 3. Results and Discussion

### 3.1. Gene Markers and Primer Sets for Freshwater Microalgae Metabarcoding

**Figure 1:**
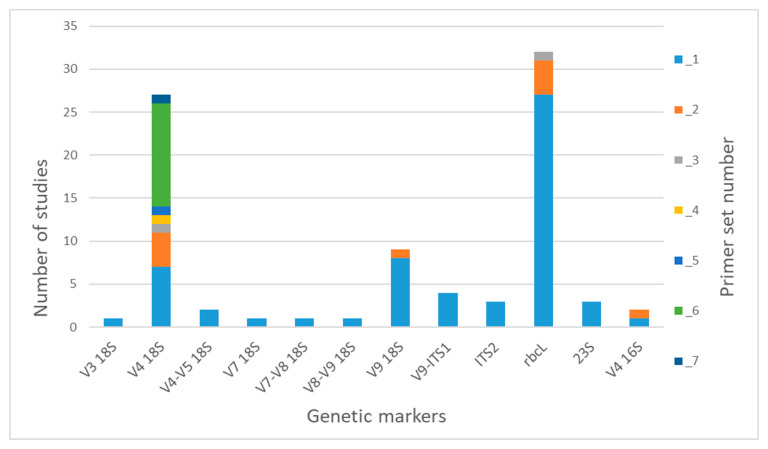
Gene markers and primer sets used for freshwater microalgae metabarcoding. The sets of primers have been assigned numbers that are listed in Table 1.

We found that 12 various genetic regions are used in the studies (Figure 1). The nuclear regions V3, V4, V7, V9 and V9-ITS1 are used for analyzing whole eukaryotic communities, as well as communities of microalgae, also focusing on individual groups of algae (dinoflagellates and diatoms [31] (Table S1)). The ITS2 region has been chosen for studying green algae s.l. (*Viridiplantae*) in a series of studies of the Antarctic region [72,73,74,105]. The plastid region *rbc*L is widely used for diatom metabarcoding, and also, primers for identifying Eustigmatophyceae have been designed and tested [97]. There are only three studies on cyanobacterial and eukaryotic algal diversity that were carried out using the universal plastid barcode 23S (Figure 1). The 16S rRNA gene has been used even less (in only two studies) as a universal marker for prokaryotes and eukaryotic algae.

Among nuclear markers, the V4 18S rRNA region is used for analyses most often (Figure 1). In the reviewed studies, we found seven options of primer sets, the most used of which was Set 6 (TAReuk454FWD1/TAReukREV3), developed by Stoeck et al. [43] (Table 1). This set is widely used in metabarcoding of both marine and freshwater eukaryotic plankton. Sets 1 (DIV4for/DIV4rev3) and 2 (M13F-D512/M13R-D978rev) are aimed at diatoms and used in seven and four studies, respectively. The remaining sets are all mentioned in only one publication each, apart from Set 7 (TAReuk454FWD1/V4r), which has been recently accepted as the standard for using environmental DNA in Finnish marine phytoplankton monitoring. The V9 18S region was chosen as a barcode in nine publications. The universal primer Set 1 (1391F/EukBr) was mostly used for the amplification of this region (in eight publications out of nine). In addition, this region and the primer set were used in a large-scale project called the Earth Microbiome Project (EMP; http://www.earthmicrobiome.org (accessed on 22 May 2023)). Set 2 (1380F(1389F)/1510R) was used in the research of a brackish lake [53].

Most of the studies on diatom metabarcoding used *rbc*L and primers Set 1 (Diat_ *rbc*L _708F_1, 2, 3/R3_1, 2, 312 bp), first suggested by Vasselon et al. [16]. The set designed by Kelly et al. [17] (*rbc*L 646F/*rbc*L 998R) for the adaptation of a DNA metabarcoding approach to ecological assessments within the Environment Agency’s routine monitoring program in the UK was used less often (4 publications out of 32).

We found only one publication each where the regions V3 18S, V4–V5 18S, V7, V7–V8 18S and V8–V9 18S were used as genetic markers. Studies on metabarcoding with the regions V9-ITS, ITS2, 23S and V4 16S all used one respective primer set (Table 1).

### 3.2. Reference Databases for Sequence Interpretation

**Figure 2:**
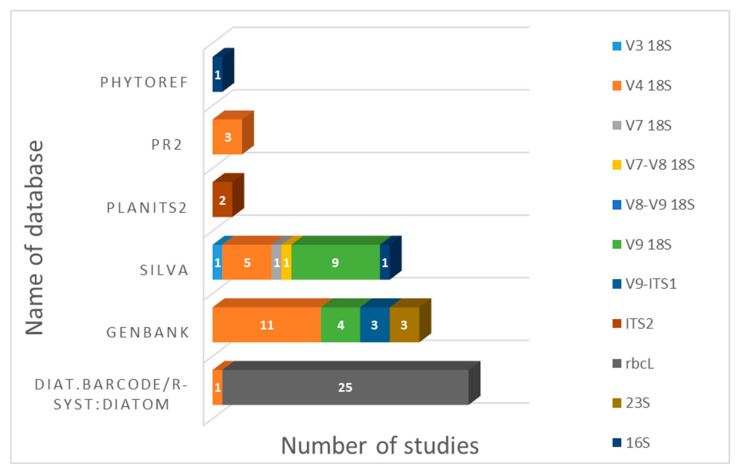
Usage of reference databases for annotating sequences in freshwater microalgae metabarcoding studies (Rsyst:diatom version v7 renamed “Diat.barcode” in February 2018 [49]).

During our literary analysis, we noticed that authors used different databases for taxonomic attributions of sequences (Figure 2). Studies on diatoms that use the *rbc*L region always use “Diat. barcode” (Rsyst:diatom database), a curated barcode library for diatoms [105] for sequence interpretation. Taxonomic attributions of sequences of various 18S rRNA (V3, V4, V7, V8, V9 and combinations) regions are usually carried out using GenBank, as well as quality-controlled databases of ribosomal RNA gene sequences such as “SILVA” [106]. The PR2 (Protist Ribosomal Reference) database—a catalog of unicellular eukaryote small subunit rRNA sequences with curated taxonomy—is used less often. In a series of studies on Antarctic green algae [72,73,74], the sequences were annotated using a recently established reference dataset PLANiTS, which included the sequences of Viridiplantae ITS1, ITS2 and entire ITS sequences, including both Chlorophyta and Streptophyta [107]. To classify the 16S reads of freshwater diatom biofilm [103], PhytoREF, a reference database of the plastid 16S rRNA gene of photosynthetic eukaryotes, was used [108].

To sum up, metabarcoding studies most often use specialized reference datasets with curated taxonomy in order to interpret the sequences acquired during a study.

### 3.3. First Works on Testing Genetic Markers on Monoclonal Microalgal Cultures Provide Insight on the Effectiveness of Amplification and the Resolution of Species Differentiation

The first studies that tested the resolution of genetic markers for species differentiation were carried out using large collections of monoclonal algal cultures. It allowed to determine the effectiveness of primers in amplifying certain regions, directly compare the variability of sequences and morphological features (including cryptic species) and establish the regions that are most suitable for further research. These studies became the basis of choosing the markers for next-generation sequencing.

One of the first tests of diatom ”barcode” genes (COI, *rbc*L, 18S and ITS rDNA) was done by Evans et al. in 2007 [109]. The study aimed to determine the effectiveness of markers in distinguishing cryptic species within the model “morphospecies” *Sellaphora pupula* agg. As a result of their analysis, the authors suggested the barcode region COI as a valuable phylogenetic marker. However, they also reported some difficulties with the amplification of this gene (a large primer set was used, sequences for *Seminavis* cf. *robusta* and for centric diatoms could not be obtained and only partial sequences were obtained for the araphid pennate diatom *Tabularia* sp.). According to the acquired data, the plastid gene *rbc*L is less variable than COI, but it supports all the phylogenetic lines of the latter. As for ITS, this barcode has a lot of variability in the length of the region, and there is also the problem of intraindividual variations. Behnke et al. [110] ”recorded three types of ITS sequences that differed at 48 positions and two indels of 50 and 4 bp” within one *Sellaphora auldreekie* isolate.

Later, Moniz and Kaczmarska [111] tested as a barcode the small ribosomal subunit (SSU, 1600 bp), a 5′ end fragment of the cytochrome c oxidase subunit 1 (COI, 430 bp), and the second internal transcribed spacer region combined with the 5.8S gene (5.8S + ITS2, 300–400 bp) on 28 species from 22 genera of diatoms. COI showed the lowest rates of amplification (only 29% of good quality DNA amplified with COI, and of those, only 30% were sequenced successfully and found to be diatom DNA). For SSU, the authors noted the highest of all three success rates in amplification and easy alignment; however, a long fragment is required for species delimitation. 5.8S + ITS2 showed a higher rate of successful amplification and sequencing (79% and 84%, respectively), as it was the most variable of the three markers, but its secondary structure was needed to aid in alignment. As a result, the 5.8S + ITS2 fragment was proposed as the best candidate for a diatom DNA barcode. In their next work, M. Moniz and I. Kaczmarska [112] confirmed the successful use of 5.8S + ITS2 for differentiating diatoms on a large selection of sequences: 618 sequences representing 114 diatoms from classes Mediophyceae and Bacillariophyceae. In particular, a 99.5% success rate in separating species was shown and a 91% success rate in separating species using a short barcode starting at the 5′ end of 5.8S and ending in the conserved motif of helix III of ITS2 (300 to 400 bp).

A search for a universal marker for diatoms was carried out by Hamsher et al. [113]. The authors assessed the following markers: ∼1400 bp of *rbc*L, 748 bp at the 3′ end of *rbc*L (*rbc*L-3P), LSU D2/D3 and UPA. As a result, *rbc*L-3P was suggested as the primary marker for diatom barcoding, since it had the power to distinguish all species and could be sequenced more easily. LSU D2/D3 could distinguish all but the most closely related species (96%). UPA showed low resolution, distinguishing only 20% of the species. Relying on the authors’ personal experiences (several copies were amplified, and the resulting sequences were different in length and unreadable), as well as the literary data, it was concluded that ITS is not a good barcode for diatoms.

The effectiveness of *rbc*L was discussed by M. MacGillivary and I. Kaczmarska [114]. A 540-bp fragment 417 bp downstream of the start codon of the *rbc*L gene was tested on a large selection of diatom taxa from classes Mediophyceae and Bacillariophyceae (381 sequences representing 66 genera and 245 species). This fragment was chosen after preliminary testing as the most variable. As a result, this fragment of *rbc*L correctly segregated 96% and 93% of the morphological congeners, respectively. The authors indicated a limitation in the resolution of biologically defined and closely related species (e.g., *Pseudo-nitzschia* and *Stephanodiscus*); using a *p* = 0.02 cut-off, only 80% of biological species were segregated. The authors noted that, with the total diversity of the diatoms (near 200,000 species), up to 40,000 species might be misidentified by their proposed *rbc*L barcode.

The effectiveness of three markers (SSU rDNA, *rbc*L and COI) for metabarcoding was tested on a mock community of diatom algae (30 strains belonging to 21 species) by Kermarrec et al. [115]. These markers are the primary ones used for the molecular identification of diatoms. The markers ITS and LSU were not considered in this study because of their high interclonal variability and the lack of available data for the establishment of reference libraries. In order to interpret the acquired sequences, reference libraries were created for each marker. Sequences from the authors’ own collection and from GenBank were included in these libraries. Gene marker *rbc*L showed the best species composition assessment of the mock community, and SSU rDNA was next (it did not differentiate the complexes *Nitzschia palea* and *Gomphonema parvulum* at the intraspecific level). COI is variable and provides high resolution, but it was not recommended for routine metabarcoding due to difficulties in amplification and low representativity of the reference library.

A large work on assessing the utility of the gene markers COI, *rbc*L, ITS, *tuf*A, UPA and 18S for freshwater green algae was done by Hall et al. [116]. They tested representatives of seven distantly related species groups from classes Chlorophyceae, Charophyceae and Zygnematophyceae (151 strains, 40 species total). As a result, the authors concluded that 18S, UPA and COI would be poor choices for a DNA barcode in green algae (18S and UPA proved insufficiently variable and COI difficult to amplify). ITS, *rbc*L and *tuf*A were sufficiently variable to distinguish most species of Chlorophyceae, but additional primers were sometimes needed for amplification. For the charophytes, *rbc*L was noted as the most suitable primer but with a remark that it was impossible to differentiate species using this marker alone.

A detailed study of within-species and between-species genetic distances for ITS region (using 81 dinoflagellate species belonging to 14 genera) showed that “…the sequence of the dominant ITS region allele has the potential to serve as a unique species-specific ‘‘DNA barcode’’ that could be used for the rapid identification of dinoflagellates...” [117]. This idea has been supported by other research done on dinoflagellates [118,119,120].

Our search criteria did not reveal any similar research on other groups of algae; however, the review by Leliaert et al. [8] showed that the sets of main markers employed for DNA-based species delimitation in Chrysophytes, Cryptophytes and Raphidophytes included nuclear markers SSU rDNA and ITS, and for Xanthophytes, they also included ITS, whereas, for Euglenophytes, the barcode markers were plastid, and nuclear SSU rDNA, LSU rDNA and ITS were not used.

Summarizing the results of the first studies concerning the search of DNA barcodes for different groups of algae, we can conclude the following: the UPA region is insufficiently variable, COI is difficult to amplify, 18S can be amplified successfully but is insufficiently variable and LSU D2/D3 cannot distinguish the most closely related species in diatoms. For diatoms, the most effective genetic marker has proven to be *rbc*L; in green algae, this region is difficult to amplify (additional primers are needed). The ITS region successfully distinguishes species of Chlorophyceae and dinoflagellates, but in diatoms, alignment is difficult, and there are problems connected with a high level of intraspecific variability [109,110]. In charophytes, ITS is difficult in amplification. This fundamental research highlights the limitations of metabarcoding and explains the instances of common species being missed while using only one marker or taxonomic attribution being limited at the genus level.

### 3.4. 18S—Choosing a Variable Barcode Region for Eukaryotes In Silico

The eukaryotic gene 18S-rRNA is used for species delimitation in almost all groups of freshwater algae [8]. It contains nine hypervariable regions (V1 to V9), each of which has been considered as a short barcode for species identification (with the exception of V6, because this region is more conserved in eukaryotes) [121] (and references in it). The question of using hypervariable regions as barcode markers for eukaryotes in silico has been discussed in several publications.

Stoeck et al. [43] provided pairwise comparisons of 7503 publicly available sequences of dinoflagellates and showed that the V4 region is less variable compared to the V9 region (the number of homopolymers per sequence is 6.8 times higher in the V4 region compared to the V9 region). On the whole, V9 detected a wider range of higher taxonomic groups than V4.

Based on an alignment of eukaryotes containing 24,793 positions from the SILVA database, the characterization of the 18S rRNA gene and the design of universal eukaryote specific primers were provided by Hadziavdic et al. [13]. To describe the nucleotide variation in the alignment, the authors used Shannon entropy values. The results suggested that the V2, V4 and V9 regions were best suited for biodiversity assessments (they yielded the highest taxonomic resolutions at cut-off values ranging 95–100% for the sequence identity). The V1 region is rather short (ca 100 nt) and contains a highly conserved core segment, and the V3 and V5 regions lack highly variable segments and are not very long. V7 has a highly variable core of approximately 20–25 nt. The V8 region is over 150 nucleotides long with variable and conserved positions interspersed across the region, with a conserved segment towards the 3′ end. The authors noted that there were no nucleotide segments of sufficient length for standard PCR along the whole gene that were entirely conserved within all eukaryotes while being absent in prokaryotes. Therefore, a single primer pair that will cover the full eukaryotic diversity and, at the same time, exclude prokaryotes cannot be designed. The authors mapped the available universal primers from the literature, as well as self-designed primers (total 100 non-degenerate eukaryote primers), and suggested two pairs of universal eukaryote-specific primers targeted to V4 (F574/R952) and V7–V8 (F-1183/R-1631) (Table 1). However, the authors noted that the coverage of eukaryotic taxa may be lower, as with the universal eukaryotic primers.

A comparative study of the validity of three regions of the 18S-rRNA gene (V1–3, V4–5 and V7–9) for the planktonic eukaryotic community was done by Tanabe et al. [121]. They showed that the V1–3 region (568 nt) has the highest variability and identification power, followed by the V7–9 region (484 nt), and the V4–5 region (415 nt) has the lowest variability. Based on in silico PCR analyses, the authors showed that the number of sequences from international nucleotide sequence databases (INSDs) such as DDBJ, EMBL and GenBank for the V4–5 region was 5–22 times higher than for V1–3 and 3–4 times higher than for V7–9. Nevertheless, the authors concluded that no significant difference was detected between the V1–3 and V7–9 regions, so the V1–3 region was suggested for the mass parallel sequencing-based monitoring of natural eukaryotic communities. Subsequently, the choice of genetic markers was limited by the use of the Illumina MiSeq platform (250–300 nt single read length, resulting in ∼450–500 nt-long combined reads with 50–150 bp overlap). Therefore, amplicons with length >500 nucleotides such as V1–3 (568 nt) were excluded [54].

Thus, based on in silico PCR analyses, it was concluded that the V1–3, V7–9 and V9 regions are more variable than V4 and V4–V5. V1–3 is too long for the Illumina MiSeq platform and cannot be used for metabarcoding. The number of sequences in international nucleotide sequence databases differs for the V4–5 and V7–9 regions.

### 3.5. 18S rRNA Gene Metabarcoding: V4 vs. V9

Several studies have been dedicated to comparing the efficiency of using the V4 and V9 regions for characterizing the diversity of eukaryotic communities.

Bradley et al. [54] examined the effect of PCR/sequencing bias of the V4 and V8–V9 regions on community structure and membership using seven microalgal mock communities consisting of 12 algal species across five major divisions of eukaryotic marine and freshwater microalgae. The authors found a critical shortcoming of the V4 primer set as used in the literature [43] and described the failed sequencing runs. The V4 region failed to reliably capture 2 of the 12 mock community members (the haptophytes *Prymnesium parvum* and *Isochrysis galbana*), whereas the V8–V9 hypervariable region more accurately represented the mean relative abundance and alpha and beta diversity. Bradley et al. [54] found that degeneracies on the 3′ end of the current V4-specific primers impacted the read length and mean relative abundance. They modified the TAReukREV3 reverse primer and suggested the V4r primer without degeneracies on the 3′ end for the subsequent sequencing (Table 1). Overall, the V4 and V8–V9 regions showed similar community representations, but their specific samples were markedly different. Therefore, the authors suggested that multiple primer sets might be advantageous for gaining a more complete understanding of community structures.

A comparative analysis of the V4 and V9 regions of 18S rDNA of the eukaryotic community of a pond [53] showed a remarkable discrepancy: the inventory of the major subdivision groups in the V9 region dataset did not correspond to that in the V4 region dataset. Eukaryotic OTUs for the V9 region were 20% more abundant than those for the V4 region at a 97% identity threshold. V9 also showed a larger diversity from the point of view of taxonomic coverage. The classes Karyorelictea, Prostomatea and Nassophorea in Ciliophora and the family Perkinsida (‘Alveolata’ group) were not detected using the V4 sequencing data, whereas they were detected using the V9 sequencing data. V4 missed Echinamoebida, Eumycetozoa and Euamoebida and green microalgae classes Chloropicophyceae, Pyramimonadophyceae and Mamiellophyceae. The authors noted “… the simultaneous application of two biomarkers may be suitable for understanding the molecular phylogenetic relationships”.

In an investigation dedicated to a eukaryotic community in anaerobic wastewater treatment systems [48], the V4 and V9 regions also detected different taxonomic groups. The authors suggested that commonly used V4 and V9 primer pairs could produce a bias in eukaryotic community analyses. The number of sequences of the amplicon library for the V9 region was almost two times larger than the number of sequences of the V4 amplicon library (340,054 vs. 180,678). The V4 region-specific primer pair showed that the dominant group was fungi. However, the V9 region-specific primer pair showed a large portion of prokaryotic sequences (bacteria and archaea accounted for 52.2% and 35.6% of the total number of sequences, respectively.) Ultimately, the authors concluded that the V9 region-specific primer pair was not suitable for the analysis of eukaryotic communities in an upflow anaerobic sludge blanket reactor, because a large number of prokaryotes sequences was detected.

It is interesting to note that similar results were obtained in a comparison of these genetic markers in marine eukaryotic communities.

In the study of a eukaryotic community of marine anoxic waters, Stoeck et al. [43] showed similar results for these regions (V4 and V9) on the diversity profiles (higher rank taxon groups that were represented by a proportion ≥1% of all unique tags in at least one of the two sets of amplicons). However, the example of dinoflagellates showed that the V4 and V9 primer pairs detected very different taxonomic profiles at the genus and family levels. The authors connected these differences with the selectivity of primers that preferentially detect different dinoflagellate subgroups. On the other hand, sets of dinoflagellate taxa represented in GenBank by V4 and V9 SSU regions overlap only partially, which could artefactually lead to apparently different taxa being detected.

A comparison of the 18S rRNA V4 and V9 regions for coastal phytoplankton communities with a focus on Chlorophyta [122] showed that the V9 region provided 20% more OTUs built at 97% identity than V4. Interestingly, the expectations were the opposite: the authors assumed that V4 as the longer region would detect more OTUs. The authors noted that both markers work “…equally well to describe global communities at different taxonomic levels from the division to the genus and provided similar Chlorophyta distribution patterns”. The authors concluded that V9 was the better choice for Chlorophyta, as it was more discriminating than V4. In the same cases for prasinophytes clade VII, V9 OTUs allowed to discriminate all subclades defined to date, while, in V4, several clades collapsed together. However, there was also an opposite example: “The V9 region of some *Chlamydomonas* is very similar to that of prasinophytes clade VII A5”. The authors emphasized the importance of the existence of reference sequences in databases, the absence of which, for instance, prevented the assessment of Dolichomastigales (Chlorophyta and Mamiellophyceae) diversity using V9. Similar results were demonstrated on marine picoeukaryotes [123], amoebae [124] and zoonotic trichomonads [125].

Piredda et al. [126] reported similar patterns for the V4 and V9 markers. The authors compared data from metabarcoding and LM approaches using the example of marine planktonic protist assemblages. For Bacillariophyta, comparable taxonomic patterns were shown between the sequence and light microscopy data, whereas, for Dinophyta, there was an overrepresentation in the sequence dataset (authors explained it by the large genome size in this group and the relationships between genome size and rDNA copy numbers). The reassuring outcome of this study was the overall comparable results of taxonomic analyses obtained with V4 and V9 on the same samples. The diatom patterns across samples were rather similar between V4 and V9 at the levels of genera and species. Due to the failure in the identification of *Pseudo-nitzschia* in the V9 sequences, the authors associated this with the smaller reference dataset available for V9.

Overall, the taxonomic composition of the eukaryotic community in the V4 datasets differed from that in the V9 dataset. V9 provided more diversity on higher taxonomic levels (supergroup and phylum), whereas the V4 region missed some important eukaryotic groups (for example, the algae classes Chloropicophyceae, Pyramimonadophyceae and Mamiellophyceae). However, in the phylogenetic analyses of eukaryotes, the V4 region has a much better resolution than the V9 region [54]. It should also be taken into account that sets of taxa represented in databases by V4 and V9 SSU regions only partially overlap.

### 3.6. Internal Transcribed Spacer Ribosomal DNA (ITS) in Metabarcoding Researches

The ITS region is the accepted DNA barcode for fungi and a strong locus for delimiting or identifying species from different algal groups, such as Chlorophyta, Dinophyceae, Chrysophyceae, Xanthophyceae and Eustigmatophyceae [7,8]. Therefore, the usage of this region for metabarcoding has positive prospects, with a high probability of identifying nucleotide sequences at the species level. We found several studies that used ITS as a barcode region. As far as we are aware, there are no metabarcoding studies that compare ITS with other markers.

The V9-ITS1 region of the 18S was chosen for the large-scale research of freshwater protists from 217 freshwater lakes across Europe [68,69,70]. The studies were aimed at identifying the diversity dynamic of the protist communities relative to the geographic distance and mountain range structures [68], centers of endemism [70] and models of interactions between the protist community and bacteria [69]. In regard to algae, the diversity of the following groups was determined in these studies: Dinophyceae, Chrysophyceae, diatoms, Cryptophyta and Viridiplantae (green algae). The same materials and the same methods were used for research on the phylogenetic and functional diversity of Chrysophyceae [71]. It was shown that Chrysophyceae are one of the most common groups in freshwater ecosystems (found in 213 out of 218 sample sites across Europe).

The ITS2 gene region is the best marker for DNA barcoding of Chlorophyta. This marker resolves major green algae lineages (some with high bootstrap support), has a high resolution for taxonomic assessment (enables the most species to be distinguished) and a high level of universality (i.e., in primers for PCR) [127] (and references in it). This region was successfully used in the first studies of the diversity of Viridiplantae (including green microalgae) in the Antarctic using the metabarcoding approach in soil and rock surfaces samples [72,128], sediments from lakes [74] and glacial ice [73]. The interpretation of sequences was carried out using the PLANiTS2 database [107], and most of the taxa were identified to the species level.

### 3.7. Gene Markers for Diatoms

Diatoms are well-known ecological indicators of aquatic ecosystems and are widely used for routine monitoring. Indexes of the water quality in rivers and lakes have been developed on the basis of diatoms and are used in EU countries (the Water Framework Directive in Europe), the USA (the National Water Quality Assessment Program in the USA), Canada, Australia and New Zealand [105,129,130,131]. Therefore, adapting the metabarcoding method for use as a tool for ecological assessment is a relevant task of modern research.

The first works on metabarcoding of freshwater diatoms suggested the V4 18S region as a candidate for a barcode marker. Zimmermann et al. [37,132] demonstrated a high correlation of the results obtained by microscopy and by metabarcoding. The authors used effective specific primers M13F-D512 and M13R-D978rev (Table 1) that were tested on non-axenic unialgal cultures of 123 taxa of Bacillariophyta (including closely related species, the genus *Sellaphora* (incl. the *Sellaphora pupula* group)) and showed that the V4 18S rRNA fragment is variable enough for taxa identification. Still there is a balance between marker variability and primer universality. The latter is important for the reproducibility of laboratory protocols. Although 18S V4 does not allow sufficient resolution for cryptic species, the authors believe that this does not matter for ecological studies, because representatives of cryptic species groups usually have similar ecological preferences.

Visco et al. [32] showed a strong similarity between the DI-CH (the Swiss Diatom Index) values inferred from microscopic and V4 18S NGS analyses of diatom communities. However, the authors noted that the interspecies variability of this barcode might change between different genera, and its effectiveness would depend on the taxonomic composition of the diatom community. The V4 resolution did not allow to unambiguously assign *Navicula* species, but it was sufficient to distinguish most of the species of *Nitzschia* and *Gomphonema*.

The *rbc*L gene marker has a wider application for studying diatom communities, and thanks to the establishment of a quality reference database Diat.barcode/R-syst:diatom [105], it can already be considered the standard for diatom metabarcoding.

A region 263 bp long (or 312 bp, including primers) and a set of primers first suggested by Vasselon et al. [16,94] (Table 1, *rbc*L primers Set 1) is used in the overwhelming majority of studies. This marker choice is based on the works of Kermarrec et al. [115,133], who compared the nuclear gene 18S and the plastid gene *rbc*L and showed that the resolution of the *rbc*L gene provides detection at the species level, while 18S is efficient at the genus level.

The resolution of the *rbc*L 312 bp marker on the level of intraspecific and cryptic diversity was successfully demonstrated by Pérez-Burillo et al. [80]. Benthic diatom samples (n = 610) were studied with a special focus on several ecologically important diatom species that are also key for the Water Framework Directive monitoring of European rivers: *Fistulifera saprophila*, *Achnanthidium minutissimum*, *Nitzschia inconspicua* and *Nitzschia soratensis*. As a result, it was shown that intraspecific and cryptic diversity can be assessed and understood through the application of DNA metabarcoding. For example, the genetic variants within *Achnanthidium minutissimum* and *Fistulifera saprophila* were detected. There was no correlation between the phylogenetic lineages and ecological preferences, which emphasized the “…necessity to work at the lowest “taxonomic” level possible”.

In a study of diatom endemism in high-altitude alpine lakes, Rimet et al. [84] showed the resolution of *rbc*L 312 bp at both the species and subspecies level. The analysis of the acquired data allowed the authors to draw important conclusions: high diversity was detected at the subspecies level, and the proportion of shared taxa equaled only 1.5% (in contrast, at the species level, the proportion of shared taxa equaled 15%); therefore, the level of endemism was very high, as the more sites were occupied by a species, the higher its intraspecific diversity. Finally, application of automated molecular species delimitation methods to *Achnanthidium minutissimum* revealed a hidden diversity of five and seven putative species, which did not appear to be monophyletic on the tree and had no geographic structuring.

A longer *rbc*L region (331 bp) was suggested as a result of large-scale research (500 benthic samples from 250 sites in England) with the aim of adopting a metabarcoding approach for ecological status assessment using diatoms [17]. The choice of region was based on an analysis of 390 sequences from a database. Eleven conservative regions of the *rbc*L gene with >96% identity were identified. These regions were used for developing primers. Variable regions were also analyzed, and four of these showed good potential for species delimitation. Consequently, primers were developed for these latter regions, and tests were conducted in order to determine the most effective region. As a result, based on its taxonomic coverage, amplicon length, primer conservation and robust performance, amplicon K (331 bp) with the primer pair *rbc*L-646F/*rbc*L-998R (Table 1) was selected for use in all downstream Illumina analyses for benthic diatoms.

An evaluation of two overlapping *rbc*L markers of 263 (312 bp, including primers) and 331 bp (common region 263 bp) was done recently by Pérez-Burillo et al. [86]. A large dataset was used for the study (1703 benthic diatom samples), and the results were thoroughly analyzed, considering (i) the effect of marker choice on taxonomic assignment, (ii) in-depth analyses on species discrepancies, (iii) comparison of the nucleotide and amino-acid variability (Shannon entropy) and (iv) effects of the marker choice on ecological status assessment. It was shown that the 331 bp marker demonstrates a higher resolution of species and infraspecific variants (some ASVs were unambiguously classified at the species level based only on the 331 bp marker: *Surirella brebissonii*, *Halamphora montana* and *H. banzuensis*). The authors noted, however, that false negatives were possible (some ASVs were classified into the same species by both markers, but the identifications could be rejected for one or the other marker because the bootstrap support values were very low (≥85); some ASVs could not be identified to the species, because they were identical to the reference sequences for more than one taxon). However, the biotic index (IPS) scores derived from both markers were very highly correlated and the choice of the 263 bp or 331 bp the *rbc*L marker had no important effects on the ecological status assessments. But the higher resolution of the longer marker may be preferable in ecological or biogeographical studies.

### 3.8. Specific Primers Targeted to rbcL Region Detected a High Diversity of Eustigmatophyceae

A high diversity of Eustigmatophyceae was found in environmental DNA samples with the help of new specific primers targeted at the *rbc*L region [97] (Table 1). The authors compared their results to previous studies concerning Eustigmatophyceae and concluded that diversity of this group was underestimated. The designed primers allowed to detect 184 ASV haplotypes that were either Eustigmatophyceae (179) or possibly Eustigmatophyceae (15), while, in previous works, representatives of this group were reported only as rare or single finds. The sensitivity of eustigmatophyte-directed *rbc*L primers was compared higher to universal eukaryotic 18S primers. The authors suggested that the employed techniques can be used for future studies of the population structure, ecology, distribution and diversity of this class.

### 3.9. Comparison of rbcL and 18S Markers for Freshwater Diatoms Biomonitoring

Inconclusive results were obtained in a study using the *rbc*L and V4 18S rRNA markers [34] for benthic diatoms biomonitoring in freshwater habitats of Northern Europe. The classes of ecological condition differed significantly depending on the used method: only 48% of samples with the 18S marker and 37.5% of samples with the *rbc*L marker had the same ecological status as with the morphological analysis. The assessment of the ecological conditions gave different results using different markers. The authors connected this with the differences in the taxonomic scope of the corresponding reference databases and primer specificity. For example, *Tabellaria flocculosa* was always detected with the *rbc*L marker and never with 18S (even though they are represented in the reference database). Barcodes for green algae were present only in the 18S dataset and were completely absent from the *rbc*L dataset. According to the authors, the amplification of green algae in some samples while using the 18S marker led to a low percentage of detected diatoms in the sample. In general, however, the *rbc*L marker generated species lists were more similar to the ones generated by the morphological approach. In the end, the authors found it difficult to recommend one marker over the other.

Similar research was conducted by Apothéloz-Perret-Gentil et al. [33]. They compared the same markers (fragment of the *rbc*L gene and the V4 region of the 18S rRNA gene) for the inference of the molecular diatom index. However, it was shown that, generally, a slightly better correlation with the morphological reference was observed with the *rbc*L marker due to the fact that it was more taxonomically resolutive, and the distinction of the diatom and other species was more accurate. As valuable advantages of the *rbc*L gene, the authors noted the primer specificity and the existence of the comprehensive curated Diat. barcode reference database [105]. The generated species lists based on *rbc*L were more exhaustive than the ones generated by the 18S marker. In the authors’ opinion, *rbc*L so far represents the ideal candidate for the implementation of metabarcoding methods for routine river monitoring.

### 3.10. A 23S rDNA Plastid Marker for Simultaneous Detection of Eukaryotic Algae and Cyanobacteria

The universal plastid amplicon (UPA) is the variable Domain V of the 23S plastid rRNA gene ∼330 bp in length. This region was proposed by Sherwood and Presting [99] as a marker for plastid-containing organisms, i.e., all lineages of eukaryotic algae and Cyanobacteria. In this research, a single pair of universal primers was designed, and it was indicated that these exact priming sequences are present only in cyanobacteria and plastids. However, comparisons with other markers showed the insufficient effectiveness of UPA. For example, Hamsher et al. [113] assessed four gene markers (COI, *rbc*L, LSU D1/D2 and UPA) for barcoding diatoms and concluded that the amplification of UPA was excellent, but this region was considerably more conserved among diatoms and distinguished only 20% of species. Hall et al. [116] reported UPA as the least variable locus in freshwater green algae. In charophytes (e.g., *Chara*, *Desmidium* and *Micrasterias*), there were difficulties with sequencing, and in the *Nitella* strains, the universal primers most often amplified a non-target region.

The low efficiency of this marker compared to 18S genes was confirmed by Cahoon et al. [10] based on a metabarcoding analysis of freshwater planktonic protists. The 18S barcode identified a much larger number of photoautotrophic genera OTUs (198) than 23S (75), from which 22 genera (9.5%) were uniquely identified by 23S and 145 (65.9%) by 18S. To our knowledge, this marker is used fairly rarely for metabarcoding studies of algae (Figure 1 and Table 1). Apart from the work of Cahoon et al. [10], 23S metabarcoding was conducted for a study of phytoplankton community structure and diversity of the aquaculture system for *Litopenaeus vannamei* [99], an examination of phytoplankton in the unique hypersaline system of Great Salt Lake’s Gilbert Bay (Salt Lake City, UT, USA) [100] and a multimarker analysis of an algal biofilm community [134]. Bonfantine et al. [103] reported failing to detect diatoms in 23S reads from stream biofilm samples. The low differentiating power of this marker should also be taken into account [117,120]; in the aforementioned studies, taxonomic attribution was done only to the genus level. 23S rRNA is also not included in the list of strong loci in use for delimiting or identifying species of algae [7,8].

### 3.11. The 16S rRNA Gene as a Marker for Simultaneous Detection of Prokaryotes and Eukaryotes

The 16S rRNA gene was first proposed as a metabarcoding marker by Eiler et al. in 2013 [101] on the basis of it being universally present in prokaryotes (including cyanobacteria), as well as in chloroplasts of eukaryotes. This enabled the simultaneous detection of prokaryotic and eukaryotic phytoplankton taxa. The authors analyzed the phytoplankton diversity from 49 lakes, including three seasonal surveys, and assessed the data using NGS and microscopy. The NGS approach detected 1.5–2 times more OTUs than there were taxa found by the microscopy approach. A more detailed comparison of taxonomic groups revealed that Heterokonta, Euglenophyta, Cryptophyta and Dinophyta were overrepresented in the microscopic biovolume dataset compared to the NGS data, whereas Cyanobacteria were proportionally overrepresented in the NGS dataset compared to microscopic biovolume data. The authors noted that Dinophyta, a major phylum in microscopic data, was poorly detected by NGS in some lakes. Discrepancies also included Euglenophyta and Heterokonta that were scarce in the NGS but were frequently detected by microscopy. The NGS approach detected a deep-branching taxonomically unclassified cluster that could not be linked to any group identified by microscopy.

Later, Huo et al. [57] opined that the chloroplast 16S rDNA gene might not be an appropriate choice for detecting eukaryotic phytoplankton diversity because of a bias toward bacteria. The common primers targeting this gene cover a wide spectrum of taxa, thus reducing the sequencing efforts aimed at phytoplankton diversity. The second problem is the endosymbiotic origin of chloroplasts in eukaryotic phytoplankton and endosymbionts retained in host cells permanently or temporarily. The authors noted that diatoms, cryptophytes and haptophytes have been reported to serve as endosymbiotic chloroplasts in diverse dinoflagellate species. “Therefore, the chloroplast 16S rDNA gene might not truly reflect host phytoplankton diversity.”

Recently, Bonfantine et al. [103] explored the potential of a standard V4 515F-806RB primer pair in recovering diatom plastid 16SrRNA sequences. PhytoREF was used to classify the 16S reads from 72 freshwater biofilm samples. Based on the Clustal nucleotide alignment, the authors confirmed the differences between eukaryotic chloroplast and prokaryotic sequences. “The Ochrophyta, and other eukaryote reads, showed high sequence conservation with no 3′ mismatches in the last 5 bases of both forward and reverse 16S v4-515F and V4-806R primers. Two mismatches to the *E. coli* 16S rRNA (GT vs. TA) were observed across all aligned non-*E. coli* 16S RNA sequences 15 bases upstream of the V4-806 primer-binding site.” More than 90% of the diatom reads in each stream biofilm sample were identified. The authors found significant beta-diversity in diatom assemblages and discrimination among river segments. In an example of the three Australian environmental 16S rRNA datasets selected from NCBI-SRA, it was shown that most of the diatom OTUs (67 out of 71) were detected in other Australian ecosystems. As a result, the authors concluded that diatom plastid 16S rRNA genes are readily amplified with the standard primer sets. “Therefore, the volume of existing 16S rRNA amplicon datasets initially generated for microbial community profiling can also be used to detect, characterize, and map diatom distribution to inform phylogeny and ecological health assessments, and can be extended into a range of ecological and industrial applications.”

Overall, the 16S rRNA marker is rarely used for the simultaneous analysis of prokaryotic and eukaryotic communities (Figure 1). Although the cost of preparing a library and the volume of data increase, researchers prefer to separate one from the other and use different regions of 16S and 18S rRNA accordingly for studying prokaryotic and eukaryotic communities [30,40,50,51,135]. Nevertheless, as shown by Eiler et al. [101] and Bonfantine et al. [103], environmental 16S rRNA datasets can yield useful information on eukaryotes, but the sensitivity and the level of taxonomic attribution in this case would be much lower than while using eukaryotic markers. It should also be considered that, according to Eiler et al. [101], Heterokonta, Dinophyta, Euglenophyta and Heterokonta are often poorly detected by NGS based on the 16S rRNA.

### 3.12. Comparing Approaches: Metabarcoding vs. Morphological Identification (Congruency between Methods)

**Figure 3:**
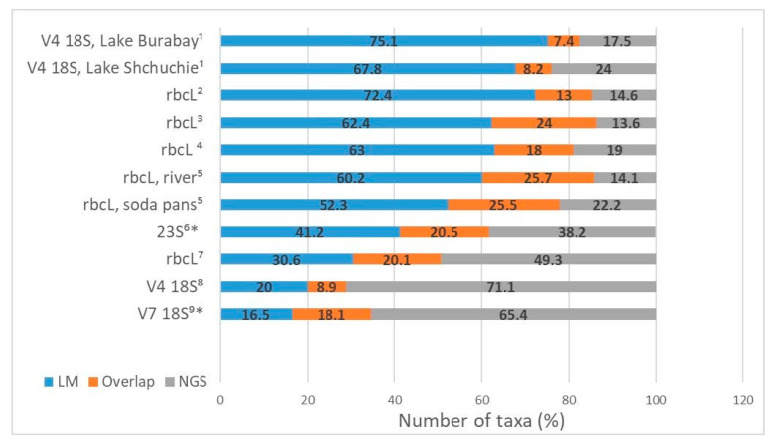
Shared taxa detected by the microscopy approach (LM), next-generation sequencing (NGS) and both (Overlap). * Publications with identification at the genus level. References: 1–[40], 2–[16], 3–[35], 4–[94], 5–[95], 6–[99], 7–[83], 8–[42], 9–[57].

Comparing the results acquired by using LM and NGS allows to reveal discrepancies between these methods and the causes of these discrepancies to determine the efficiency of the amplification of the chosen genetic markers and to identify problems in bioinformatical processing and taxonomic attribution. Every single work that compared the morphological and molecular approaches for studying the diversity of algal communities indicated a significant difference in the resulting taxonomic lists. The number of taxa detected by both approaches falls between 7.4 and 25.7% (Figure 3).

Some studies have shown that diversity detected by NGS is much higher than that found in LM. In a research of diatom diversity, Zimmermann et al. [132] reported that about 2.5 times more taxa were found by the NGS approach (263 taxa vs. 102 taxa in LM). In an example of studying benthic diatoms, Bailet et al. [34] showed that the metabarcoding method using the 18S marker revealed 27% more taxa than the morphological method and 38% more taxa using the *rbc*L marker. As a result of a study on epiphytic diatoms, Borrego-Ramos et al. [83] showed that metabarcoding detected more taxa than LM (49.3% vs. 30.6%), and only 20.1% of the taxa were concordant when comparing both methodologies. In an investigation of phytoplankton, Huo et al. [57] reported that metabarcoding using the 18S V7 gene marker detected 3.5 times more OTUs than the number of morphospecies revealed by morphological identification. The same results were shown in the studies of phytoplankton conducted by Groendahl et al. [42] based on the 18S V4 gene marker: the metabarcoding method detected 71.1% of the total taxa number, whereas LM identification found only 20% (Figure 3).

In contrast, other investigations have shown that the morphological approach allows to identify a greater diversity [16,35,40,90,95,99] (Figure 3). Using the example of diatoms, it has been shown that most of the species identified by LM are not represented in a database, including the special curated diatoms database Diat.barcode. Visco et al. [32] revealed that the GenBank database only covers 46% of the morphospecies found in microscopic analyses. Vasselon et al. [16] and Vasselon et al. [90] reported that 68% and 82% of morphological species are not represented in the database. Duelba et al. [91] revealed that 60% and 32% of taxa detected by LM in riverine and soda pan samples, respectively, are not recorded in the database. Bailet et al. [34] pointed out that only 15.4% of all Fennoscandian taxa are represented in the 18S database and 17.8% in the *rbc*L database. A comparative analysis of freshwater phytoplankton communities in two lakes conducted by Malashenkov et al. [40] showed that the NGS of 16S and 18S rRNA amplicons adequately identified phytoplanktonic taxa only at the genus level, while the species composition obtained by microscopic examination was significantly larger (67.8% and 75.1% vs. 24% and 17.5% by NGS, respectively) (Figure 3).

The challenges of taxonomic reference databases in metabarcoding analysis were recently summarized in a review by Keck et al. [136]. The authors discussed in detail the following problems: (i) mislabeling, (ii) sequencing errors, (iii) sequence conflict, (iv) taxonomic conflict, (v) low taxonomic resolution, (vi) missing taxa and (vii) missing intraspecific variants.

Here, we briefly summarize and supplement the list of reasons that explain discrepancies in the results obtained by using microscopic and metabarcoding methods on algae. These should all be taken into account while interpreting the results.

The challenges in metabarcoding analysis:

1. Gap in the reference database [16,27,32,34,38,46,62,82,90,99], etc.
2. The natural intraspecific and intragenomic variabilities of the barcoding marker (single taxon has multiple genotypes at the barcoding region, and members of that taxon might cluster into different Molecular Operational Taxonomic Units (MOTUs)) [35].
3. Cryptic diversity—a single morphological species can represent different genetic groups (e.g., diatoms *Sellaphora pupula*, *Pinnularia borealis*, *Hantzschia amphioxys* and *Nitzschia inconspicua* and species of *Stichococcus*, *Coccomyxa*, *Chlorokybus*, *Cryptomonas,* etc.) [78,137,138,139,140,141,142].
4. MOTU richness can be artificially inflated through technical errors at different steps of sample processing during amplification and sequencing [35].
5. The MOTU delimitation approach influences the richness estimation and interpretation [35] (and references in it) (assessment of the bioinformatics pipelines provided in [14,15]).
6. Complete absence of amplification on the whole due to a mismatch of the primer set used. For example, Salmaso et al. [27] did not find any species belonging to the Euglenales in the HTS results (with universal eukaryotic primers (TAReuk454FWD1 and TAReukREV3) for V4 18S), although they were present in LM. Hanžek et al. [66] reported that the taxa that contributed most to the biomass (*Actinotaenium/Mesotaenium* sp. and the species *Cosmarium tenue*, *Pantocsekiella comensis*, *Sphaerocystis schroeteri* and *Synedropsis roundii*) were not identified by eDNA metabarcoding (V9 18S region was amplified using the universal primer pair 1391F and EukB). Proeschöld and Darienko [140] noted that, although *Stichococcus*-like organisms are widely distributed in almost all habitats, they are not recorded in environmental studies based on HTS approaches, because the V4 or V9 regions of the SSU contain introns that obstruct amplification. Groendahl et al. [42] reported that *Monorhaphidium* sp., *Selenastrum* sp. and *Trachelomonas* sp. detected using the morphology-based approach were not identified by the metabarcoding approach, despite the fact that all three genera are included in the reference database.
7. Uncertainties and lack of sensitivity of reference databases for the selected DNA markers [27].

The challenges related to morphological identification:

1. Diatom extracellular skeletons are counted in LM even if they come from dead cells. The valves of dead cells can be transported from locations other than the target assemblage. Metabarcoding will not detect these dead cells [35,78].
2. The proportion of live diatoms found in environmental samples varies greatly, ranging from 2 to 98% [35].
3. Small-celled species and pico-sized cells are often overlooked or underestimated by the morphological approach. For example, the valves of *Fistulifera saprophila* tend to dissolve during sample processing, which can explain why this species is often missed during morphological identification [78,80,82,99].
4. LM misidentification (false LM positives) [27,78,99].
5. Differences in the detection limits of the two methods: morphological and molecular approaches do not give the same insight into communities of algae and, therefore, do not have the same detection capacity for species [27,92,99].
6. The different sample volumes settled for microscopy and metabarcoding [143].
7. Underlying units used for microscopy (individual cells) and those used for metabarcoding (ASV sequences) are quite different, making direct comparisons imperfect [24,143].
8. A short barcode gene fragment may have limited the taxonomic resolution [143]. For example, the resolution of the V4 18S region does not allow to unambiguously identify some species of *Navicula* [32]. For the V7–9 18S marker, a lack of intergenus taxonomic resolution was found (the MOTUs matched multiple genera, e.g., *Alexandrium pseudogonyaulax* and *A. hiranoi*, *Chaetoceros neogracile* and *C. curvisetus* and *Thalassiosira eccentrica* and *T. antarctica*) [144]. In some *Chlamydomonas*, the V9 region is very similar to that of prasinophytes clade VII A5 [122].

Thus, each method is imperfect and has its limitations. For better understanding and interpretation of the metabarcoding results, studies that use both methods are still relevant. The filling of databases with identified nucleotide sequences with metadata will, in the future, greatly improve the quality of taxonomic attribution and metabarcoding data interpretation. Nevertheless, as pointed out by Bailet et al. [14], “…the longer-term goal should be to break free from the preconceptions we have brought with us from careers based around light microscopy and to recognize HTS data as distinct”.

## 4. Conclusions

Metabarcoding has already been accepted as an alternative (faster and more economical) method to the traditional microscopy method for the ecological assessment and monitoring of freshwater bodies of water, rivers and seas based on microalgae. Protocols, technical guidelines or standards for eDNA monitoring are developed and/or approved in many countries [145,146,147,148,149] (Table S2). The results of our review show that, currently, there is no one perfect marker for identifying microalgae across the whole diversity. Among the most popular genetic barcodes for freshwater metabarcoding, we can highlight the nuclear regions V4 and V9 18S rRNA (which allow to determine the composition of auto- and heterotrophic eukaryotes) and the region of the plastid gene *rbc*L (for diatoms). The regions ITS1 and ITS2 might be underestimated, but they show good potential for usage as a microalgae barcode; they can be easily amplified with the standard primers and are variable enough to identify sequences to the species level.

The choice of marker is determined by the focus of the study, for example, a certain group or groups of algae (a specific marker and/or primers can be used, like a marker for diatoms or Eustigmatophyceae); a screening of eukaryote diversity (universal markers V4 and V9 18S are suitable) or an assessment of interactions between different groups of organisms (a set of markers for various groups of prokaryotes or eukaryotes, invertebrates and vertebrates should be used). Some studies have used a multimarker approach. For example, Wolf and Vis [134] used four markers (V9 18S, *rbc*L, 23S and V4 16S rDNA) for identification of an algal biofilm community. To investigate the dynamic evolution of multitrophic communities (bacterial and eukaryotic) under ecohydrological changes, Liang et al. [52] used three markers: V3–V4 16S rRNA (for bacteria), the V4 region of 18S rRNA and COI (for eukaryotes) and universal primers. Robinson et al. [89] used multimarker metabarcoding to study diatoms and macroinvertebrate indicators (*rbc*L and COI markers, respectively). In a large-scale study of benthic macroinvertebrates and diatoms in rivers, Seymour et al. [46] compared the 18S universal molecular marker and molecular markers that target traditional biomonitoring groups (*rbc*L, 12S and COI). For sequence identification, the most comprehensive databases for each marker were used: NCBI for COI and 12S, Silva for 18S and Diat.barcode for *rbc*L. Such investigations are tied to processing a massive amount of data for different groups of organisms (thousands of sequences are involved). Therefore, it is complicated to verify all taxonomic attributions obtained during automatic processing. Looking through the Supplemental Materials for the latest study [46], we noticed several misidentifications. For example, using the *rbc*L marker (eDNA method), several representatives of different classes were assigned to the class Bacillariophyceae: *Dictyosphaerium*, *Lobosphaera* (Trebouxiophyceae), *Kirchneriella*, *Oedogonium*, *Pandorina*, *Volvox*, *Pediastrum* (Chlorophyceae), *Tribonema* (Xanthophyceae), *Lagynion* (Chrysophyceae), etc. This shows that there are faults in the databases and confirms once more the necessity of verification for taxonomic lists obtained by NGS, since these mistakes can lead to incorrect descriptions of communities and inaccurate conclusions.

Below, we briefly summarize the main advantages (+) and disadvantages (−) of markers that are used for freshwater microalgae metabarcoding.

***rbc*L.**

“+” widely used for diatom metabarcoding, distinguishes between taxa at the species and intraspecies levels; a high-quality curated reference database for taxonomic attribution Diat.barcode [105].

“−” extremely heterogeneous in green algae; does not have a set of universal primers [127]; only diatoms are identified well.

Notes: the majority of studies use the region with the length of 263 bp (312 bp including primers) and a complicated set of primers (three forward primers and two reverse primers) suggested by Vasselon et al. [16,90] (Table 1, *rbc*L primers Set 1). However, recently, it has been shown that a longer region of 331 bp (common region 263 bp, proposed by Kelly et al. [17,18], primers set *rbc*L 646F-*rbc*L 998R) has a higher resolution for species and intraspecific variants [86].

**V4 18S.**

“+” widely used for metabarcoding of marine and freshwater eukaryotes; successfully amplified by a universal primer set. The V4 region (named pre-barcode) was designated as the starting point for the identification of protists in the International Barcode of Life Consortium Project (iBOL, http://www.ibol.org/ (accessed on 22 May 2023) and the Protist Working Group (ProWG) [150]. Provides an understanding of molecular phylogenetic relationships.

“−” compared with V9 18S, misses haptophytes [54], many groups of heterotrophs and green algae from the classes Chloropicophyceae, Pyramimonadophyceae and Mamiellophyceae [53]. The V4 region is less variable compared to the V9 region. Often does not differentiate species.

**V9 18S.**

“+” widely used for marine and freshwater eukaryotes metabarcoding; successfully amplified by a universal primer set; was chosen to amplify eukaryotes in the global project “The Earth Microbiome Project” (EMP; http://www.earthmicrobiome.org (accessed on 22 May 2023)); V9 is more variable than V4; provides more OTUs and diversity on higher level taxa (supergroup and phylum).

“−” a short region (96 bp–134 bp [36]; sometimes does not differentiate species.

Notes: the V4 and V9 regions detect different taxonomic profiles at the genus, family and macro-taxa levels. Both markers are recommended for a more complete understanding of community structures.

**ITS** (V9-ITS1 or ITS2 regions are used for metabarcoding).

“+” is a strong locus for some algae (Chlorophyta, Dinophyceae, Eustigmatophyceae and Xanthophyceae) [7]; sufficiently variable; allows to differentiate species; amplified by a universal primer set. A specialized curated reference dataset “PLANiTS” [107] exists for interpreting data, including ITS1, ITS2 and entire ITS sequences of Viridiplantae.

“−” for diatoms, this barcode has a great variability in the length of region and a problem in intraindividual variation [110,113].

**23S (UPA)**.

“+” algal-specific markers focused on plastid-containing eukaryotic algae and Cyanobacteria; sufficiently amplified by a universal primer set.

“−” a conserved region; low resolution; identifies taxa only to the genus level or higher; is not a strong locus for algae.

**16S** (V3–V4 and V4 regions is used for metabarcoding).

“+” focused on the chloroplasts of eukaryotes and prokaryotes (Cyanobacteria); sufficiently amplified by a universal primer set; can be used to simultaneously detect prokaryotes and eukaryotic algae.

“−” biased towards bacteria in the community; might not accurately reflect the phytoplankton diversity due to the endosymbiotic origin of chloroplasts; is not a strong locus for algae.

Notes: is used very rarely for the simultaneous complex analysis of prokaryotes and eukaryotic algae.

In general, it should be taken into account that every marker will demonstrate a different image of the community that depends on successful amplification of the chosen region. The identification of the taxonomic composition and the level of taxonomic attribution depends on the region variability and the quality of the reference databases.

## Figures and Tables

**Table 1:** Different available markers and primer sets used in bulk metabarcoding studies of freshwater algae.

Number of Primer Set	Gene Region	Target Group	Primer Name		Primer Sequence 5′ to 3′ (Primer Author Reference)	Forward/Reverse	References	PCR Cycling
	V3 18S	Eukaryotes			ATTAGGGTTCGATTCCGGAGAGG	forward	[30]	n.d.
					CTGGAATTACCGCGGSTGCTG	reverse		
					[31]			
1	V4 18S	Diatoms	DIV4for:		GCGGTAATTCCAGCTCCAATAG	forward	[14,32,33,34,35,36]	94 °C—2 min (35 cycles: 94 °C—45 s, 50 °C—45 s, 72 °C—1 min), 72 °C—10 min
			DIV4rev3		CTCTGACAATGGAATACGAATA	reverse		
					[32]			
2	V4 18S	Protist, Diatoms	M13F–D512		TGT AAA ACG ACG GCC AGT ATT CCA GCT CCA ATA GCG	forward	[10,37,38,39]	94 °C—2 min, (5 cycles: 94 °C—45 s, 52/54 °C—45 s, 72 °C—1 min), (35 cycles: 94 °C—45 s, 50/52 °C—45 s, 72 °C—1 min), 72 °C—10 min.
			M13R–D978rev		CAG GAA ACA GCT ATG AC GAC TAC GAT GGT ATC TAATC	reverse		
					[37]			
3	V4 18S	Eukaryotes	F574		GCGGTAATTCCAGCTCCAA [13]	forward	[40]	95 °C—5 min, (25 cycles: 98 °C—1 min, 98 °C—20 s, 51 °C—20 s, 72 °C—12 s), 72 °C—1 min.
			1132r		CCGTCAATTHCTTYAART [41]	reverse		
4	V4 18S	Eukaryotes			AATTCCAGCTCCAATAGCGTATAT	forward	[42]	98 °C—30 s, (30 cycles:98 °C—10 s, 59 °C—30 s, 72 °C—30 s), 72 °C—10 min.
					TTTCAGCCTTGCGACCATAC	reverse		
					[42]			
5	V4 18S	Eukaryotes	F574		GCGGTAATTCCAGCTCCAA	forward	[13]	PCR *in silico*, Tm 55.3
			R952		AAG ACG ATC AGA TAC C	reverse		
					[13]			
6	V4 18S	Eukaryotes	TAReuk454FWD1		CCAGCA (G/C)C(C/T)GCGGTAATTCC [43]	forward	[10,27,44,45,46,47,48,49,50,51,52] *	94 °C—5 min, (15 cycles: 94 °C—30 s, 53 °C—45 s, 72 °C—1 min), (20 cycles: 94 °C—of 30 s, 48 °C—45 s, 72 °C—1 min), 72 °C—10 min.
			TAReukREV3		ACTTTCGTTCTTGAT(C/T)(A/G)A [43]	reverse		
			V4 forward		CCAGCAGCCGCGGTAATTCC [43] modfied primers from [43]	forward	[53]	
			V4 reverse		ACTTTCGTTCTTGATTAA [43] modfied primers from [43]	reverse	[53]	
7	V4 18S	Eukaryotes	TAReuk454FWD1		CCAGCA (G/C)C(C/T)GCGGTAATTCC [43]	forward	[54]	95 °C—5 min, (10 cycles: 94 °C—30 s, 57 °C—45 s, 72 °C—1 min), (15 cycles: 94 °C—30 s, 47 °C—45 s, 72 °C—1 min), 72 °C—10 min.
			V4r		ACTTTCGTTCTTGAT [54] modfied primers from [43]	reverse		
	V4–V5 18S	Eukaryotes	563f		GCCAGCAVCYGCGGTAAY	forward	[41,55,56]	
			1132r		CCGTCAATTHCTTYAART	reverse		
					[41]			
	V7 18S	Eukaryotic phytoplankton community	960F		GGCTTAATTTGACTCAACRCG	forward	[57]	Two-step tailed PCR. Round 1: 95 °C for 3 min, (15 cycles: 95 °C—1 min, 55 °C—1 min, 72 °C—1 min), 72 °C—10 min (260 bp). Round 2: 98 °C—30 s, (10 cycles—98 °C—10 s, 55 °C—30 s, 72 °C—30 s), 72 °C—5 min.
			NSR1438		GGGCATCACAGACCTGTTAT	reverse		
					[58]			
	V7–V8 18S	Eukaryotes	F-1183		AAT TTG ACT CAA CAC GGG	forward	[13]	The annealing temperature of 52 °C
			R-1631		TAC AAA GGG CAG GGA CGT AAT	reverse		The annealing temperature of 59.1 °C
					[13]			
	V8–V9 18S	Eukaryotes	V8f 1422		ATAACAGGTCTGTGATGCCCT [54]	forward	[30,54]	95 °C—3 min (25 cycles: 98 °C—20 s, 65 °C—15s и 72 °C—15 s), 72 °C—10 min.
			1510R		GCCTTGCCAGCCCGCTCAG (eukaryotic) [59]	reverse		
1	V9 18S	Eukaryotes	1391F		GTACACACCGCCCGTC [60]	forward	[10,48,61,62,63,64,65,66]	92 °C—3 min, (30 cycles: 45-s—92 °C, 1-min—57 °C, 1.5-min—72 °C.) 10 min—72 °C.
			EukBr		TGATCCTTCTGCAGGTTCACCTAC [67]	reverse		
2	V9 18S	Eukaryotes	1380F		CCCTGCCHTTTGTACACAC (eukaryotic)	forward	[53]	94 °C—3 min, 30 cycles: 94 °C—30 s, 57 °C—60 s, 72 °C—90 s), 72 °C—10 min 94 °C 10 min, (35 cycles: 94 °C—40 s, 58 °C—25 s, 72 °C—30 s), 72 °C—10 min.
			1389F		TTGTACACACCGCCC (universal)	forward		
			1510R		CCTTCYGCAGGTTCACCTAC (eukaryotic)	reverse		
					[59]			
	V9-ITS1	Protist			GTACACACCGCCCGTC	forward	[68,69,70,71]	98 °C—3 min, (35 cycles: 98 °C—30 s, 52 °C—75 s, 72 °C—60 s), 72 °C—10 min.
				ITS2_Dino; 10%	GCTGCGCCCTTCATCGKTG	reverse		
				ITS2_broad; 90%	GCTGCGTTCTTCATCGWTR	reverse		
	ITS2	Chlorophyceae		ITS3	GCATCGATGAAGAACGCAGC	forward	[72,73,74]	n.d.
				ITS4	TCCTCCGCTTATTGATATGC	reverse		
					[75]			
1	*rbc*L	Diatoms		Diat_ *rbc*L _708F_1	AGGTGAAGTAAAAGGTTCWTACTTAAA	forward	[14,16,22,33,34,36] ** [76,77,78,79,80,81,82,83,84,85,86,87,88,89,90,91,92,93,94,95]	95 °C—15 min, (30–40 cycles: 95 °C—45 s, 55 °C—45, 72 °C—45 s) (final extension).
				Diat_ *rbc*L _708F_2	AGGTGAAGTTAAAGGTTCWTAYTTAAA	forward		
				Diat_ *rbc*L _708F_3	AGGTGAAACTAAAGGTTCWTACTTAAA	forward		
				R3_1	CCTTCTAATTTACCWACWACTG	reverse		
				R3_2	CCTTCTAATTTACCWACAACAG	reverse		
					[16]			
2	*rbc*L	Diatoms		*rbc*L 646F	ATGCGTTGGAGAGARGTTTC		[17,46,86,96]	95 °C—15 min, (32–35 cycles: 95 °C—20 s, 55 °C—45 s, 72 °C—60 s),72 °C—5 min.
				*rbc*L 998R	GATCACCTTCTAATTTACCWACAACTG			
					[17]			
3	*rbc*L	Eustigmatophyceae		EU *rbc*L 500FA	GGNCGYGTWGTDTWYGAAGGT	forward	[97]	The annealing temperature of 53.5 °C
				Eustig *rbc*L-R900	CACCWGCCATACGCATCC	reverse		
					[97]			
	23S	Protist	p23SrV_f1		GGA CAG AAA GAC CCT ATG AA	forward	[10,98,99]	94 °C—2 min, (35 cycles: 94 °C—20 s, 55 °C—30 s, and 72 °C—30 s) 72 °C—10 min.
			p23SrV_r1		TCA GCC TGT TAT CCC TAG AG	reverse		
					[100]			
1	V3–V4 16S	Freshwater phytoplankton	341F		CCTACGGGNGGCWGCAG	forward	[101]	95 °C—5 min, (25 cycles: 95 °C—40 s, 53 °C—40 s and 72 °C—1 min) 72 °C—7 min.
			805R		GACTACHVGGGTATCTAATCC	reverse		
2	V4 16S	Diatom plastid	515F		GTGYCAGCMGCCGCGGTAA [102]	forward	[103]	94 °C for 3 min, (30–35 cycles: 94 °C—30 s, 53 °C—40 s, 72 °C—1 min), 72 °C—5 min.
			806R		GGA CTA CHV GGG TWTCTA AT [104]	reverse		

* in [48] forward named V4_1F, in [52] primers named 547F/V4R; ** in [36] used only Diat_ rbcL _708F_2 and R3_1 primers.

## Data Availability

Not applicable.

## Supplementary Materials

The following supporting information can be downloaded at: https://www.mdpi.com/article/10.3390/biology12071038/s1: Table S1: Reference list used in the analyses. Table S2: Barcode markers and primers used in the guidelines and standards for metabarcoding of various groups of algae and cyanobacteria.
